# Single and Multiple Stimuli-Responsive Polymer Particles for Controlled Drug Delivery

**DOI:** 10.3390/pharmaceutics14020421

**Published:** 2022-02-15

**Authors:** Aida López Ruiz, Ann Ramirez, Kathleen McEnnis

**Affiliations:** 1 Chemical and Materials Engineering Department, New Jersey Institute of Technology, Newark, NJ 07102, USA; al568@njit.edu; 2 Biomedical Engineering Department, New Jersey Institute of Technology, Newark, NJ 07102, USA; adramire@umd.edu

**Keywords:** stimuli-responsive, drug delivery, polymer particles

## Abstract

Polymers that can change their properties in response to an external or internal stimulus have become an interesting platform for drug delivery systems. Polymeric nanoparticles can be used to decrease the toxicity of drugs, improve the circulation of hydrophobic drugs, and increase a drug’s efficacy. Furthermore, polymers that are sensitive to specific stimuli can be used to achieve controlled release of drugs into specific areas of the body. This review discusses the different stimuli that can be used for controlled drug delivery based on internal and external stimuli. Internal stimuli have been defined as events that evoke changes in different characteristics, inside the body, such as changes in pH, redox potential, and temperature. External stimuli have been defined as the use of an external source such as light and ultrasound to implement such changes. Special attention has been paid to the particular chemical structures that need to be incorporated into polymers to achieve the desired stimuli response. A current trend in this field is the incorporation of several stimuli in a single polymer to achieve higher specificity. Therefore, to access the most recent advances in stimuli-responsive polymers, the focus of this review is to combine several stimuli. The combination of different stimuli is discussed along with the chemical structures that can produce it.

## 1. Introduction 

Controlled release of drugs is a growing field with many challenges to overcome. Many drugs are hydrophobic, which limits their bioavailability. Other drugs, such as chemotherapy drugs, are very toxic and ideally should only be released once, at the tumor site. Polymeric nanoparticles have been extensively studied as a platform for specific and controlled drug delivery, and can potentially solve these problems. Polymeric nanoparticles for drug delivery have been proven to increase the circulation time, enhance drug accumulation at the tumor site in cancer therapies, reduce the side effects of drugs, and improve tolerance [1]. Biocompatibility and biodegradability are two other factors that make polymers so favorable [2]. Many polymers have been extensively used in the field of drug delivery [3,4]. The most commonly used biodegradable polymers are poly(lactic-co-glycolic) acid (PLGA) and poly (ε-caprolactone) (PCL). Whereas the most common non-biodegradable polymers are poly (methyl methacrylate) and polyacrylate [4].

**Figure 1:**
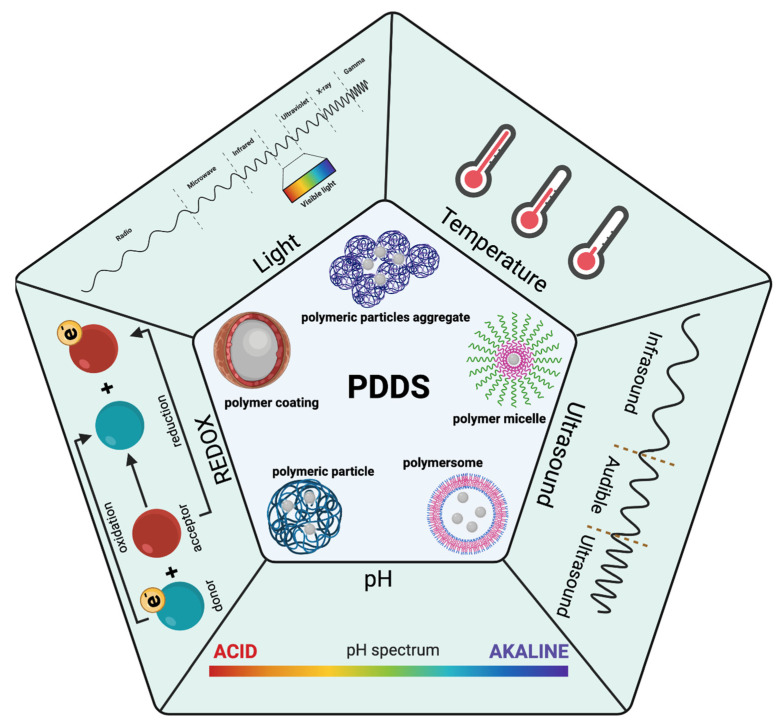
A schematic representation of stimuli that can trigger drug release using particle drug delivery systems (PDDS) based on polymers. Created in Biorender.com, accessed date (2 September 2022).

External or internal stimuli can trigger the controlled release of drugs. Internal stimuli can be considered thermal, pH, and redox potential, while external stimuli consist of light and ultrasound as represented in Figure 1. Moreover, dual-responsive polymers enable drug delivery methods and therapeutic efficacy to be fine-tuned. Previous reviews have investigated stimuli-responsive polymers and their applications in drug delivery; however, the field has been developing quickly and there have been many advances in recent years. Multiple stimuli polymers have emerged as the new trend to achieve finer control of the release of drugs and avoid side effects [5,6,7,8]. While there are reviews focusing on the use of stimuli-responsive polymers for targeting or imaging purposes [9,10,11,12,13], this review will specifically summarize the progress in stimuli-responsive polymers as particles for controlled drug release, with a focus on the recent advances in the field. As there are already several recent reviews discussing the role of hydrogels in drug delivery [14,15,16,17,18,19], including the use of nanogels as particles for drug delivery, we will not include hydrogel polymer particles in this discussion. Instead, we will discuss the attributes that make a polymer responsive to stimuli, how they are used as drug delivery particles for controlled drug release, and possible future uses. 

## 2. Single Stimuli

Stimuli-responsive polymer particles have become important in the field of drug delivery due to the potential for controlled release. Several stimuli can be used for this purpose. Table 1 presents a brief summary of the different stimuli that we will discuss in this review with some examples of the active parts needed within a polymer to achieve the desired stimuli response. Further discussion will be provided in the following sections for each stimulus. 

### 2.1. Internal Stimuli 

#### 2.1.1. pH-Responsive 

It is well known that different parts of the body have different pH values, especially in the gastrointestinal tract (GI), where the pH gradient varies dramatically [32]. However, the pH gradient is not just limited to the GI tract; different pH’s exist inside the cell itself. For instance, lysosomes have a pH of 4.5–5, endosomes 5.5–6, Golgi apparatus 6.4, and cytosol 7.4 [33]. One of the most important differences in pH can be observed between tumors (pH 6.5–6.8) and normal tissue (pH 7.4) [34]. This change in pH is due to a phenomenon known as the Warburg effect [35,36]. In this phenomenon a discrepancy in pH between healthy tissue and cancerous tissue is observed due to the rapid proliferation of cancer cells which decreases the blood supply, limiting the supply of oxygen and nutrients. The limited oxygen decreases the process of phosphorylation by the cells, forcing cells to take energy from glycolysis producing lactic acid, thereby decreasing the pH of that area. Based on the Warburg effect many studies have focused on polymeric nanoparticles sensitive to pH [35,36,37,38,39]. 

**Figure 2:**
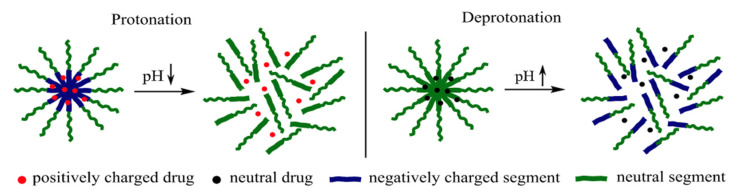
A schematic illustration of drug release from a polymer micelle. Protonation (**left**) or deprotonation (**right**) destroys the polymer micelle [43]. Reprinted from Saudi Pharmaceutical Journal, 28, M. Alsehli, Polymeric Nanocarriers as Stimuli-Responsive Systems for Targeted Tumor (Cancer) Therapy: Recent Advances in Drug Delivery, 255–265, Copyright (2020), with permission from Elsevier.

Drug-loaded polymeric nanoparticles with pH-sensitive functional groups can alter their density of charges in response to a variation in pH. This mechanism is based on the hydrophobicity of the nanoparticles as a result of protonation or deprotonation [40]. For example, co-polymer micelles can release a drug in response to pH changes as we can see in Figure 2 [41,42,43]. Palanikumar et al. synthesized polymeric nanoparticles with a functionalized membrane of acid-triggered peptide (ATRAM) [44]. ATRAM peptide has a pKa of 6.5, which gives it a high specificity for use in the acidic microenvironment of tumors. 

Another approach that can be used to make particles pH-responsive is the incorporation of cleavable bonds. The most important cleavable bonds are imine, hydrazone, hydrazide, oxime, and (di)methyl maleate. Table 2 shows a list of these cleavable bonds that can be incorporated into polymers with the relevant pH for drug delivery [45,46].

**Figure 3:**
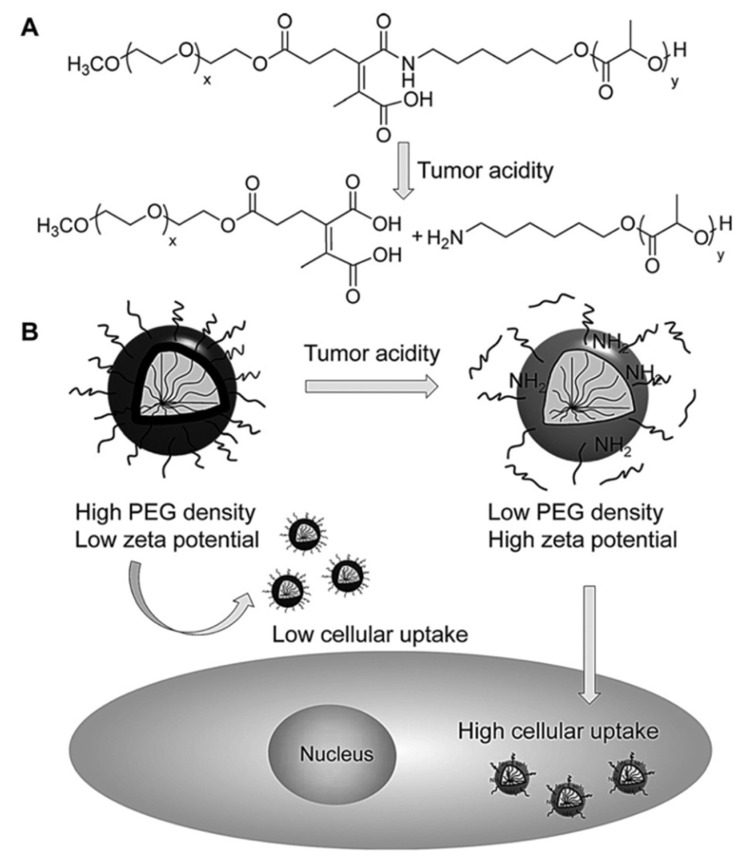
(**A**) Chemical structure and cleavable bond in acidic conditions. (**B**) A schematic illustration of the uptake mechanism of particles based on pH [21]. Reproduced with permission from C. Y. Sun et al., Angewandte Chemie—International Edition, published by John Wiley and Sons, Copyright 2016.

For instance, poly(L-histidine)-b-poly(ethylene glycol) (PH-PEG) combined with poly(L-lactic acid)-b-poly(ethylene glycol) (PLA-PEG) has been studied for tumor targeting [47]. The advantage of this system is the sharp transition between a stable and an unstable drug delivery system. It is non-ionized and hydrophobic at pH 7.4, but ionized and hydrophilic at pH 6.6 [48]. Zhang et al. produced a nano-carrier that is pH-responsive by using an imine bond [20]. In another example of pH-responsive polymers, Sun et al. synthesized polymeric nanoparticles of poly(D, L-lactide) (PDLLA) and poly(ethylene glycol) (PEG) which were linked by a (di)methyl maleate group [21]. In a weak acidic environment, the PEG dissolves, promoting endocytosis of the particles and the release of the drug [21]. The acidic pH at the tumor site triggers the cleavable bond, decreasing the PEG density and increasing the uptake of the particles by the cells (Figure 3). 

#### 2.1.2. Redox Potential-Responsive

Drug delivery systems for cancer and gene therapy are advantageous when they degrade directly in the nucleus and the cytosol of the cell while maintaining their stability in the extracellular environment [49]. Many redox processes occur in the intracellular environment, such as NADP^+^/NADPH, O_2_/O_2_^−^, and glutathione (GSH) [50]. Specifically, GSH has attracted interest in the drug delivery field. GSH’s chemical name is γ-L-glutamyl-L-cysteinyl-glycine, and it is a peptide composed of glycine, cysteine, and L-glutamic acid [51]. GSH’s concentration is used in drug delivery due to the abrupt concentration change between the intracellular (1–10 mM) and the extracellular environment (1–10 μM) [52,53,54] (Table 3). Nevertheless, GSH concentration in tumor tissue has been found to be four-fold higher than healthy tissue in mice, making GSH level a good trigger for drug delivery systems [55,56]. However, the cancer environment changes between different types of cancer. For example, in brain tumors, GSH concentration has been found to be between 0.5–3 mM [57]. Gamcsik et al., categorized many different cancer tissues and the difference in GSH levels compared to healthy tissue [58]. However, due to the high variability between the studies, the numbers have not been included in Table 3. Nevertheless, there is a general trend towards using increased levels of GSH in cancer tissue as a trigger for drug delivery systems. 

**Figure 4:**
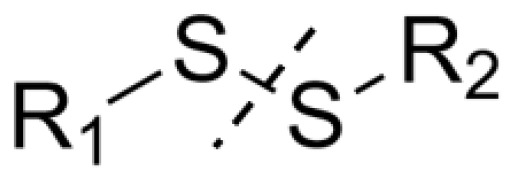
Disulfide bond responsive to redox potential (GSH).

The design of drug delivery systems sensitive to redox potential can be very versatile, and the use of polymers for these kinds of conformations is very popular [59]. One technique used to create degradable polymeric micelles involves using amphiphilic copolymers with a disulfide bond connecting the two blocks [60,61,62]. In a study by Sun et al., polymer micelles were used to deliver doxorubicin. Micelles were synthesized by using a graft copolymer of poly(acrylic acid)-g-poly(ethylene glycol) (PAA-g-PEG) which contains a disulfide bond [63]. By adding this disulfide bond micelles remained assembled until they found reductive conditions that could break the bond. Another approach for GSH-responsive particles is the incorporation of a GSH-responsive crosslinking agent in the core or the shell of the micelle [64]. The mechanism of how these polymeric micelles disassemble is based on the reduction of the disulfide bond in the polymer by the interaction with GSH, Figure 4 [65]. The micelle destabilization can shift the hydrophobic/hydrophilic balance promoting the fragmentation of the polymer into monomers, releasing the drug [66].

**Figure 5:**
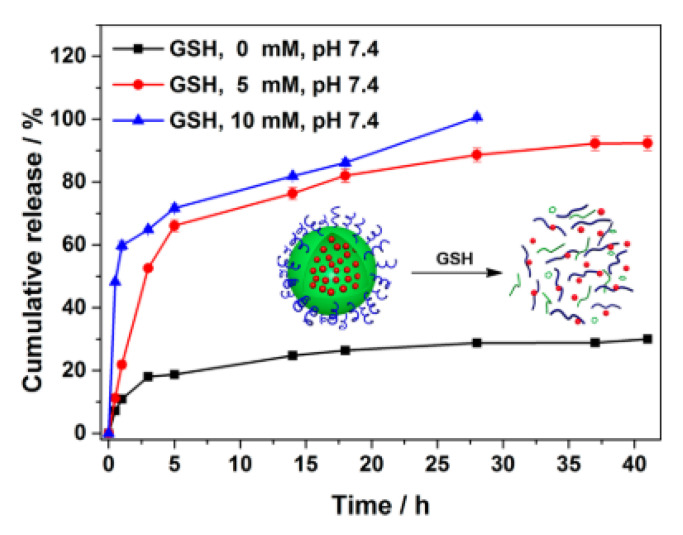
Doxorubicin release from polymeric particles at different concentrations of GSH, at the same temperature [22]. Reprinted with permission from Xu et al. ACS Biomaterials Science and Engineering **2015**, *1* (7), 585–592. Copyright 2015 American Chemical Society.

Xu et al. synthesized a triblock copolymer, with a disulfide bond [22]. Doxorubicin (an anticancer drug) was encapsulated in the polymeric system. The GSH concentration gradient was used as a delivery trigger to achieve specificity for cancer cells. By combining the enhanced permeability and retention effect (EPR) with the GSH gradient, particles delivered the drug to cancer cells. Figure 5 shows how the increase of GSH triggers the release of doxorubicin from the particles. The highest concentration of GSH achieved the fastest release of the drug. 

#### 2.1.3. Thermo-Responsive

Temperature is one of the most investigated triggers for stimuli-responsive drug delivery systems. The temperature stimulus can be internal or external. Several studies have highlighted an increase in temperature at pathological sites and tumors because of the abnormal blood flow, a high rate of cell proliferation, and metabolic activity. These temperature differences between healthy and tumorous tissue can be used as a trigger for drug delivery systems [47]. External temperature can also be applied to activate the delivery of a drug. For instance, hyperthermia can be used as a cancer treatment where the temperature increases to 45 °C at the tumor site, damaging and killing cancer cells [67]. 

**Figure 6:**
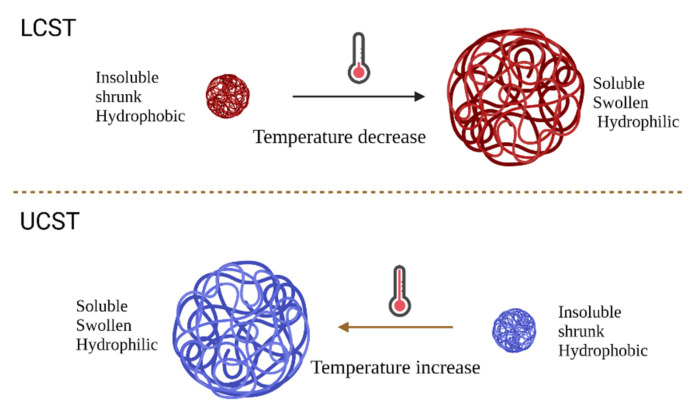
A schematic representation of LCST and UCST concepts and the polymer properties. Created in Biorender.com, accessed date (9 February 2022).

Many different materials can be used that are temperature-responsive. However, temperature-sensitive polymers are one of the most well-known materials. These polymers change their structure from a shrunken to a swollen form, in response to temperature change. The characterization of these polymers is made by the upper critical solution temperature (UCST) or the lower critical solution temperature (LCST) [59,68,69,70]. The change in the polymer conformation is activated by reaching one of those temperatures, leading to either swelling or shrinking as shown in Figure 6. 

**Figure 7:**
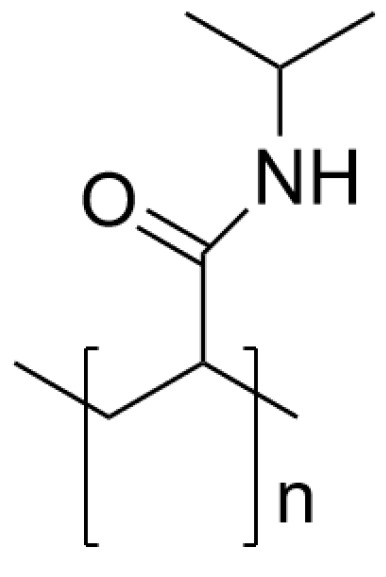
Chemical structure of PNIPAM.

The first polymer studied of this kind was poly(N-isopropyl acrylamide) (PNIPAM) Figure 7. This polymer attracted the attention of researchers due to its biocompatibility and corresponding LCST of around 32–33 °C in water, which is close to the temperature of the human body [71,72]. The LCST of the polymer can be changed by shifting the hydrophilic/hydrophobic balance by coupling it with another polymer. It has been proven that hydrophilic compounds make the LCST behavior of the polymer disappear; therefore, by changing the ratio of hydrophilic compounds the LCST can be shifted [73]. If the comonomer used is hydrophobic, it increases the LCST. If the comonomer is hydrophilic, the LCST will decrease [73,74].

**Figure 8:**
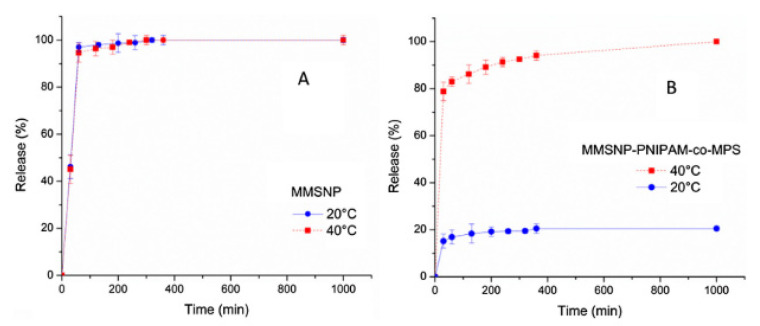
Ibuprofen release curves (**A**) nanosilica particles. (**B**) PNIPAM grafted nanosilica particles at 20 °C (blue) and 40 °C (red) [75]. Reprinted from Journal of Colloid and Interface Science, 544, M. E. Peralta et al., Synthesis and in vitro testing of thermoresponsive polymer-grafted core-shell magnetic mesoporous silica nanoparticles for efficient controlled and targeted drug delivery, 198–205, Copyright (2019), with permission from Elsevier.

Polymers responsive to temperature have emerged in biomedicine, as a potential targeted drug delivery system. Peralta et al. synthesized a temperature-responsive nanocarrier to deliver magnetic mesoporous silica nanoparticles, based on PNIPAM [75]. Nanosilica is a porous material that can be used to deliver drugs (in this case ibuprofen), and the combination with PNIPAM on the surface of the particles prevents the release of the drug at low temperatures (Figure 8). The drug release from the particles was tested at two different temperatures, 20 °C, and 40 °C, without the grafted polymer on the surface (Figure 8A) and with the grafted polymer on the surface (Figure 8B). When the polymer was not used, the drug was released immediately, with no difference between the temperatures; however, by grafting PNIPAM to the surface of the particles the release increased from 20% at a temperature of 20 °C to 80% at a temperature of 40 °C initially, and at 40 °C a final release of almost 100% of the drug was achieved. 

**Figure 9:**
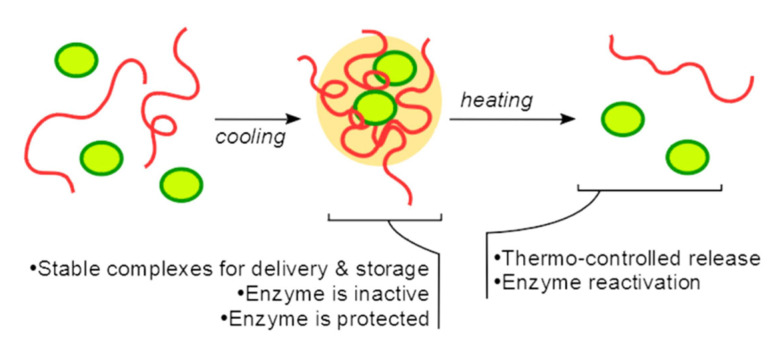
A schematic representation of an enzyme delivery system based on PNAGA [79]. Reproduced with permission from P. I. Semenyuk et al., Polymers; published by MDPI, 2021.

LCST polymers are the dominant temperature-responsive polymers in drug delivery applications; however, UCST polymers have been gaining more importance in recent years [23,76]. Compared to commonly used LCST polymers, there are fewer polymers that exhibit a UCST response [77]. Lin et al. synthesized a thermo-sensitive nanocarrier based on a UCST response for doxorubicin using the insoluble fraction of multi-L-arginyl-poly-L-aspartate (iMAPA) [78]. Additionally, iMAPA was crosslinked with hyaluronic acid (HA) to achieve selectivity to the receptors of malignant tissue. iMAPA-HA exhibits a phase transition in aqueous solutions becoming soluble at high temperatures with a UCST response. Semenyuk et al. proposed the use of poly(N-acryloyl glycinamide) (PNAGA), a UCST-responsive polymer soluble at high temperatures [79]. Figure 9 describes the technology proposed to deliver an enzyme based on a UCST thermo responsive polymer. 

### 2.2. External Stimuli 

#### 2.2.1. Light-Responsive 

Drug carriers that are responsive to light are attractive for drug delivery as the spatiotemporal release of the encapsulated material can be controlled. Many physical and chemical processes can be triggered by the radiation of a specific wavelength. Functional groups sensitive to this kind of interaction have the ability to break cleavage bonds, switch the electrostatic charge, or change the chemical conformation from cis to trans [80]. Polymers incorporating these functional groups can be used as light-responsive drug delivery systems [33,81,82].

The safe use of light in medicine is conditional on the wavelength of the light itself. Certain wavelengths can go deeper into the body but damage healthy tissue at the same time. Therefore, the use of far-UV light (a wavelength shorter than 200 nm) should be excluded from these treatments due to its potential hazard. Long-UV lasers (200–400 nm), however, can leave both the drug and tissue intact while releasing the drug from the polymer [83,84]. Visible light (400–700 nm) can also be used as a trigger, but these wavelengths are only suitable for topical treatments due to their limited penetration depth [85]. Finally, NIR radiation (750–1000 nm) has the advantage of penetrating deeper into the tissue and being benign [86]. 

UV and NIR light are, therefore, the most suitable wavelengths for light-responsive drug delivery particles. An example of a drug delivery system using polymers sensitive to both UV and NIR is the research by Liu et al. which used polymer micelles for the encapsulation of a drug [87]. In this study, dextran was combined with 2-diazo-1,2-naphthoquinone (DNQ) which is a photo-triggered group activated by interaction with UV light. However, in this study, they proved that the DNQ group can also be triggered by using NIR light which is safer than UV. When the radiation is applied, the DNQ changes charge, resulting in a change of the polymer from amphiphilic to hydrophilic, allowing delivery of the drug [87].

**Figure 10:**
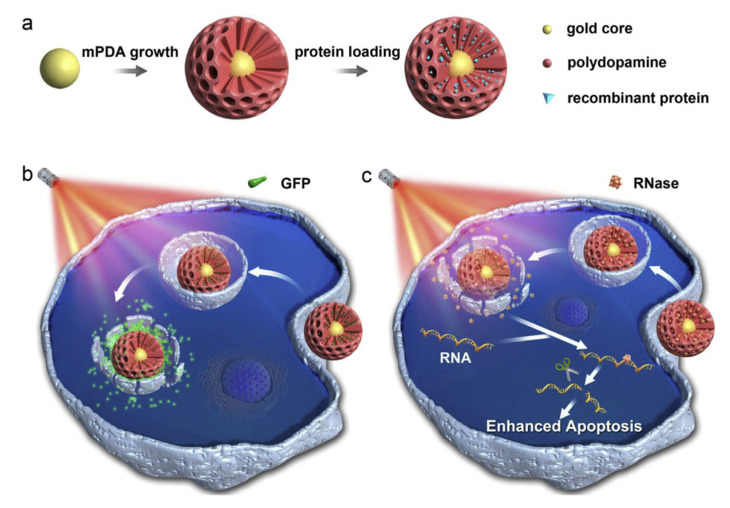
A schematic representation of (**a**) protein loading into mPDA matrix, (**b**) release of GFP protein based on NIR, and (**c**) release of RNase based on NIR [92]. Reprinted from Biomaterials, 238, D. Wu et al., Mesoporous Polydopamine with Built-in Plasmonic Core: Traceable and NIR Triggered Delivery of Functional Proteins, 119847, Copyright (2020), with permission from Elsevier.

Polydopamine (PDA) is a biopolymer used for drug delivery due to its biocompatibility, easy polymerization on the surface of particles, and its NIR-sensitive properties [88,89,90,91]. PDA exhibits a strong NIR absorption, which allows for the controlled release of encapsulated particles when irradiated with a laser at 750–1000 nm [25,26]. Wu et al. delivered proteins by attaching proteins in a mesoporous PDA delivery system [92]. By applying NIR to the system, two different types of proteins were released (Figure 10). 

#### 2.2.2. Ultrasound-Responsive

Ultrasound is high-frequency sound waves (greater than 20 kHz) produced by mechanical oscillations. Ultrasound has been used in medical applications frequently because it is a non-invasive technique that can penetrate centimeters deep into the tissue. It also has the ability to focus on a single point with high intensity. Therefore, the use of ultrasound-responsive polymers for drug delivery has recently been of interest to researchers [93]. 

High intensity focused ultrasounds (HIFU) can focus on a very small area; therefore, historically it has been used as a tumor treatment. Nevertheless, nanocarriers based on polymers responsive to ultrasound are beginning to be developed [94,95]. Disulfide bonds (S-S) are mechano-labile weak bonds that respond rapidly to HIFU, improving the ultrasound response of polymeric nanocarriers [96,97]. For example, in the research by Li et al., a block copolymer of polylactic acid (PLA) and polyethylene glycol (PEG) with a disulfide bond (PLA-S-S-PEG) was synthesized for drug delivery purposes [27]. Nanoparticles were then obtained by self-assembly of the copolymer, including a central disulfide linkage to promote sensitivity to HIFU. 

**Figure 11:**
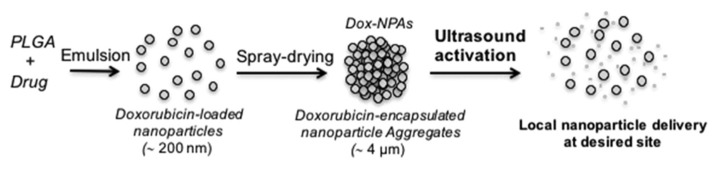
A schematic representation of PLGA carriers with ultrasound release [28]. Reprinted from Biomaterials, 139, A-L. Papa et al., Ultrasound-Sensitive Nanoparticle Aggregates for Targeted Drug Delivery, 187–194, Copyright (2017), with permission from Elsevier.

Papa et al. produced nanoparticle aggregates based on polylactic-co-glycolic acid (PLGA), to carry doxorubicin [28]. The use of ultrasound triggers the separation of the aggregates, releasing the drug into the desired area of the body (Figure 11). For the sonication process, the particles were exposed to ultrasound for 3 min with an intensity of 2.2 Watt/cm^2^. After the sonication process, size and aggregate distribution were characterized using diffraction light scattering and scanning electron microscopy. After applying ultrasound, the particles disintegrated into either single particles or smaller aggregates.

#### 2.2.3. Others

The previously mentioned single stimuli are those most commonly used in polymers for drug delivery; however, other stimuli can be implemented such as magnetism and shear force. 

Magnetism could be used as an external stimuli. A magnetic PDDS has the capability of targeting a disease site and releasing the drug when a magnetic field is applied. Several studies have combined metallic nanoparticles with polymers for this purpose. The use of a polymer helps with the compatibility of the particles, can incorporate an active target, and increase the circulation time [29,30,98,99,100]. However, the magnetic properties of these systems is achieved by the metallic nanoparticles, such as iron as was used in the studies by Cao et al. [29] and García-García et al. [30] They both used polymers as a coating on the iron nanoparticles to achieve better biocompatibility and targeting. 

Shear stress is a type of mechanical force that is interesting as a target for PDDS because it is associated with blood flow. Shear stress is commonly used as a diagnostic tool for cardiovascular diseases. Normal shear stress in arteries is 10–70 dyn cm^−2^ and 1–6 dyn cm^−2^ in veins, while for cardiovascular pathologies or hemorrhages it increases up to 100 dyn cm^−2^ [101]. Therefore, this difference can be used as an internal stimulus for PDDS. Some micelles and polymersomes have been studied due to their ability to deform their shape and release the drug under specific shear conditions. Rifaie-Graham et al. synthesized polymersomes that change shape with shear stress, thereby releasing the cargo in high shear stress conditions [31]. Shen et al. prepared micelles that are responsive to ROS production and shear stress to treat atherosclerosis, which is a type of cardiovascular disease [102]. However, most research on shear stress-responsive polymers has focused on hydrogel nanoparticles because of their flexibility [101]. 

## 3. Combination of Various Stimuli for Polymers 

Based on the type of environment and the response needed, different multiple-response polymers can be synthesized: pH/temperature, pH/redox, temperature/redox, enzyme/pH, temperature/light [103], light/redox, double pH, and temperature/pH/redox (Table 4).

Dual-responsive nanoparticles or micelles are synthesized by means of a block copolymer [125,126,127]. Block copolymers function similarly to surfactants or dispersants. These are molecules with short chains or hydrophilic and hydrophobic components, that form micelles with a hydrophobic core and a hydrophilic outer shell. In solution, block copolymers exist as individual polymer chains. However, once the critical micelle concentration (CMC) is reached, they start to form micelles [128]. In some cases, block copolymers can also form nanoparticles through kinetically controlling factors such as temperature, solvent contents, and pH. For nanoparticles to form, the CMC should be <10^−3^ wt% with a free energy change greater than 5 kT [129].

### 3.1. pH/Temperature-Responsive Polymers

pH/thermo-responsive polymers are the most widely studied dual-responsive polymers [78,110,111,112,128]. Similar to the individual pH-responsive polymers and temperature-responsive polymers, these dual-responsive polymers allow for a much more specific and targeted environment to activate the polymer. Often, dual-responsive polymers will be formulated by conjugating a pH-sensitive polymer to a thermo-sensitive polymer [130]. However, some have used a mixture of the two different classes of sensitive polymers [131]. The most common building block for thermo-responsiveness is poly(*N*-isopropylacrylamide) (PNIPAAm) [132]. This particular polymer can go from a water-soluble state to a water-insoluble state through an LCST transition. The building blocks for pH-responsiveness are often polymers such as weak acids, acrylic acids, poly[2-(diisopropylamine)ethyl methacrylate] (PDPA), and chitosan [113,131,133,134]. Once mixed, they follow the same process of a normal block copolymer to create micelles or nanoparticles.

pH and temperature-responsive polymers are frequently proposed for potential cancer therapies since the tumor environment has an increased temperature and a decreased pH [135,136,137]. In the research of Zhang et al., the effectiveness of nanoparticles made from a block copolymer of thermo-responsive hydrophilic poly(N-isopropylacrylamide-co-acrylic acid) [P(NIPAM-co-AAc)] and a hydrophobic polycaprolactone (PCL), was explored [134]. [P(NIPAM-co-AAc)] is a common polymer used for thermo-sensitive applications and PCL was chosen for its good drug encapsulation properties. This study showed that the nanoparticles released the encapsulated drug much faster at higher temperature and lower pH conditions, as are commonly seen in the tumor environment [134]. 

**Figure 12:**
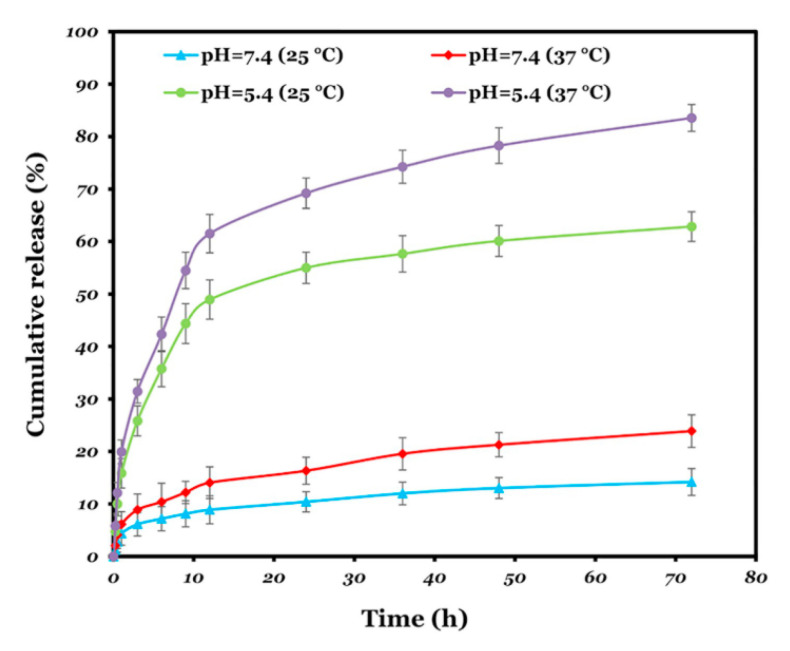
The cumulative release of doxorubicin: blue: pH 7.4 and 25 °C, green: pH 5.4 and 25 °C, red: pH 7.4 and 37 °C, and purple: pH 5.4 and 37 °C [114]. Reprinted from Materials Science and Engineering C, 108, A. Pourjavadi et al., pH and thermal dual-responsive poly(NIPAM-co-GMA)-coated magnetic nanoparticles via surface-initiated RAFT polymerization for controlled drug delivery, 110418, Copyright (2020), with permission from Elsevier.

Zheng et al. created nanoparticles using another pH and thermo-responsive copolymer consisting of poly(methacrylic acid) (PMAA) and poly(N-isopropylacrylamide) (PNIPAM) [115]. PMAA is sensitive to pH while PNIPAM is sensitive to temperature. These nanoparticles were loaded with doxorubicin (DOX), a common chemotherapy drug. Through experimentation, it was found that these particles released the DOX in acidic environments. This phenomenon was due to the electrostatic attraction between DOX (positively charged) and the polymer (negatively charged). This interaction prevented the release at neutral pH. However, the protonation of the carboxylic groups of the polymer at acidic pH weakened the interaction between DOX and the polymer, allowing for the release of the drug. The release rate was observed to be even faster when the temperature was increased above the LCST. Pourjavadi et al. used N-isopropylacrylamide (NIPAM) co-polymerized with glycidyl methacrylate (GMA), a common monomer that contains an epoxy ring, to form a copolymer of poly(NIPAM-co-GMA) (PNG) [114]. This combination of polymers provides a pH and thermo-sensitive release (Figure 12). The decrease of the pH combined with the increase in temperature to a physiological level produces a higher cumulative release, which is selective for body temperature and the pH of the endosomes. 

### 3.2. pH/Redox-Responsive Polymers 

Because redox reactions and differences in pH occur naturally in the body, these two stimuli are very appealing for drug delivery applications [133]. These types of polymers have been created for a myriad of applications, such as enhancing drug delivery and tumor cell uptake, creating a faster drug release rate within the cytoplasm and nucleus, and further stabilizing the stability of nanoparticles in vivo [116,133]. 

Bahadur et al. conjugated polyethylene glycol and cyclo(Arg-Gly-Asp-d-Phe-Cys) (cRGD) peptide to poly(2-(pyridin-2-yldisulfanyl)ethyl acrylate) (PDS) to create an RPDSG polymer [138]. Nanoparticles were created with this copolymer and DOX was encapsulated inside the nanoparticles. To induce a redox reaction, varying amounts of GSH were used in the experiment. It was found that the concentration of GSH within the extracellular fluid is less than 0.01 mM and is 1–11 mM intracellularly. After experimentation in different pH values and with different concentrations of GSH, the DOX release rate was found to be much slower at higher pH values. Under acidic conditions, the ester bonds of PDSG can be hydrolyzed to produce a faster release rate than at neutral pH, and therefore a faster release rate was achieved at pH 5.5 than at pH 7.4. Moreover, the amount of DOX released was observed to increase with a higher concentration of GSH. Mahmoud et al. took advantage of the characteristic inflammation caused by infections, cancer, or other diseases as inflamed tissues have a decreased pH as well as having reactive oxygen species present [139]. Mahmoud et al. synthesized polymeric nanoparticles that incorporate a thioether moiety into the polymer backbone [139]. In this study, they created environments that simulated healthy tissue and infected tissue with differences in pH and redox potential. It was found that the particles subjected to a pH of 5 in the presence of H_2_O_2_ were the only ones to disperse and degrade. 

**Figure 13:**
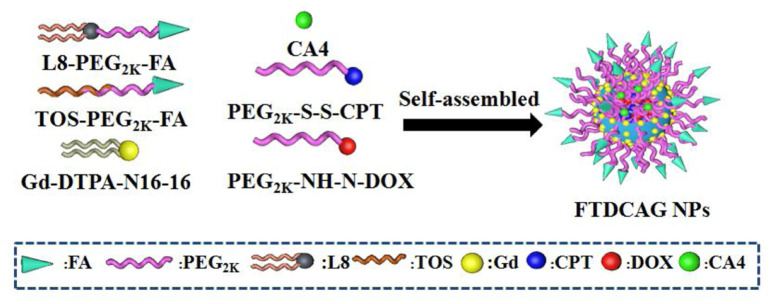
A schematic illustration of the configuration of the nanocarrier, based on double sensitive polymers with NH-H bonds and S-S bonds [121]. Reprinted from Colloids and Surfaces B: Biointerfaces, 205, N. Wang et al., A Traceable, GSH/PH Dual-Responsive Nanoparticles with Spatiotemporally Controlled Multiple Drugs Release Ability to Enhance Antitumor Efficacy, 111866, Copyright (2021), with permission from Elsevier.

In recent years, the combination of GSH concentration and pH has gained importance in the drug delivery field [117,118,119,120,122,140]. Wang et al. created dual-responsive polymeric nanoparticles based on pH and GSH concentration to deliver multiple drugs in cancerous environments [121]. The disulfide bond connecting poly(ethylene glycol) (PEG) and camptothecin (CPT), a chemotherapeutic drug, allows for the release at high concentrations of GSH (Figure 13), while the NH-N bond between PEG and doxorubicin (DOX), another chemotherapeutic drug, allows for the breaking of the hydrazine bond in acidic environments. 

### 3.3. Double-pH-Responsive Polymers 

Not only can polymers be made with responses to different stimuli, but they can also be fabricated to respond to the same stimuli but at different values. Polymers like this respond to stimuli similar to an “AND” logic gate [141]. The second event will only occur once the first event has happened. Double pH-responsive polymers are an example of this type of technology. A polymer capable of responding to two different pH values, PPC-Hyd-DOX-DA, was synthesized by Du et al. and made into DOX encapsulated nanoparticles [142]. This nanoparticle changes its surface charge from negative to positive when exposed to the pH of a tumor environment (~6.8). This change in surface charge encourages cellular internalization by the tumor cells. Once inside the endosome, the pH (~5.0) triggers DOX release within the cell [142]. This technique helps ensure that drugs targeted for tumors are specifically within the site before subsequent release.

Another example of a dual pH-responsive polymer is poly([2,2′-(propane-2,2-diylbis(oxy))bis(ethane-2,1-diyl) diacrylate]-*co*-[hexane-1,6-diyl diacrylate]-4,4′-trimethylene dipiperidine), (poly-β-aminoester ketal) [141]. When the pH is decreased, the tertiary amines in the backbone of this polymer are protonated, switching the polymer from hydrophobic to hydrophilic. This then leads to an increase in water uptake which causes bulk dissolution, which then triggers ketal hydrolysis causing surface degradation. These particles are stable at physiological pH but degrade at a pH of 5, subsequently releasing the contents of the nanoparticle. 

### 3.4. Multiple-Stimuli-Responsive Polymers

**Figure 14:**
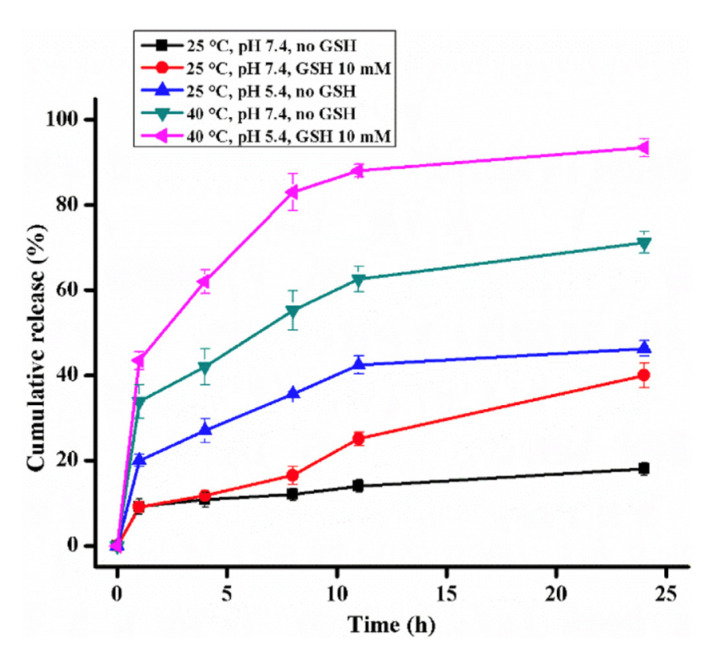
The cumulative release of doxorubicin under different conditions [105]. Reprinted from Reactive and Functional Polymers, 154, P. Poddar et al., Synthesis of a New Triple-Responsive Biocompatible Block Copolymer: Self-Assembled Nanoparticles as Potent Anticancer Drug Delivery Vehicle, 104679, Copyright (2020), with permission from Elsevier.

There has been a trend in recent years to incorporate the potential for many stimuli to trigger the drug release by a carrier to a specific disease site [90,106,107,109,123,124]. Poddar et al. synthesized a triple-stimuli-responsive polymer to achieve the release of a drug under the conditions of pH 5, 40 °C, and GSH ≥ 10 mM [106]. In this study, they synthesized two different polymers, 2-(2-((4-(hexyloxy)benzyloxy)carbonyl)ethylthio)ethyl acrylate (HBCEEA), which is sensitive to pH, and the copolymer of N-isopropyl acrylamide (NIPA) and poly(ethylene glycol methyl ether acrylate) (PEGMA), which is sensitive to temperature and redox potential. The combination of these polymers creates the triple-responsive polymer poly[HBCEEM-b-(NIPA-r-PEGMA)] (PHNP) [105]. The drug release from the polymer is much faster in the presence of all three stimuli, as shown in Figure 14.

**Figure 15:**
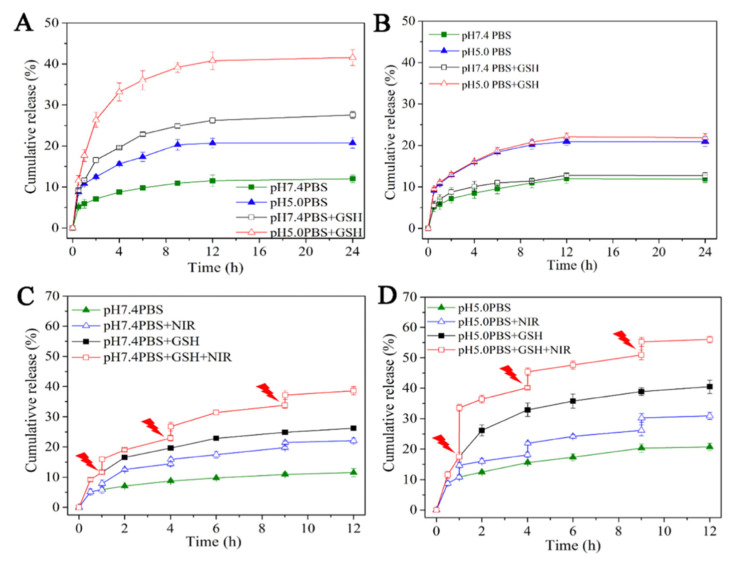
The cumulative release (**A**) MSN-PDA, (**B**) MSN-S-S-PDA, (**C**) MSN-S-S-PDA NIR pH 7.4, and (**D**) MSN-S-S-PDA NIR pH 5 [108]. Reprinted from Materials Science and Engineering C, 105, W. Lei et al., Polydopamine-Coated Mesoporous Silica Nanoparticles for Multi-Responsive Drug Delivery and Combined Chemo-Photothermal Therapy, 110103, Copyright (2019), with permission from Elsevier.

Lei et al. used mesoporous silica as the nanocarrier for doxorubicin and coated the particles with polydopamine [108]. As previously discussed, polydopamine (PDA) is highly sensitive to NIR, and with the incorporation of a disulfide bond, the particles became responsive to pH and GSH as well, achieving a multi-stimuli-responsive drug carrier. As shown in Figure 15A, mesoporous silica-disulfide bond-polydopamine (MSN-SS-PDA) and in Figure 15B, mesoporous silica-polydopamine (MSN-PDA), the incorporation of a disulfide bond increases the release rate when exposed to a low pH and high GSH. Moreover, when combined with NIR the highest cumulative release rate is observed at acidic pH combined with GSH (Figure 15D) as opposed to the neutral pH with GSH (Figure 15C), proving the multi-stimuli nature of the particles [108]. The use of an acidic pH degrades the polydopamine that coats the silica particles, allowing for a faster release of the drug. 

Furthermore, Zhang et al. synthesized a quintuple-stimuli-responsive nanocarrier based on the self-assembly of an amphiphilic diblock copolymer [104]. The conjugation of poly(2-methacry-loyloxyethyl ferrocenecarboxylate)-(5-propargylether-2-nitrobenzylbromoisobutyrate)-poly(dimethylaminoethyl methacrylate) (PMAEFc-ONB-PDMAEM), allows for the release of the drug based on temperature, pH, light, oxidation, and reduction. 

## 4. Conclusions and Future Research 

Stimuli-responsive polymer particles have become a trend in the drug delivery field due to the potential to trigger the release of drugs at specific sites, owing to changes in the environment. Specifically, in cancer research, stimuli-responsive polymer particles have become important because of the great divergence between the environment of healthy tissue and cancer tissue. In this review, many of the different stimuli that can be used to trigger the release of drugs have been studied and discussed. The current trend in stimuli-responsive PDDSs is to combine two or more stimuli. We explored the recent combinations that have been studied such as pH/temperature, pH/redox, light/pH, etc., as summarized in Table 4. Taking a more synergistic approach to the use of polymers in PDDSs, a combination of different stimuli would increase the specificity of delivery and maximize the dosage release at the tumor site. 

Currently, only simple polymeric drug delivery systems, such as PLGA particles, are available commercially. There is a significant opportunity in the market for more complex drug delivery systems such as those using responsive polymers. A few startup companies exist that are exploring the potential of stimuli-responsive particles for drug delivery applications; however, there are many challenges to overcome in taking these products to market. For instance, although using stimuli-responsive polymer particles has many advantages for drug delivery, not many have been tested in vivo. In fact, the combination of multiple stimuli in particles has not been tested in clinical trials at all, and only a few have been used in animal studies [102,143,144]. The increase in the complexity of multiple stimuli particles creates a significant hurdle in terms of the practical application of these particles in animal studies and eventually clinical trials. In addition, the stringent requirements as to reproducibility of particles in drug delivery systems will require fastidious production methods. Therefore, more research is needed to address the complexity in producing multiple stimuli particles, as this complexity hinders the commercial application of these types of particle systems. 

## Figures and Tables

**Table 1:** Single stimuli-responsive polymers.

Stimuli	Active Part	Examples	Ref.
pH	Cleavable bonds	Imine bond: HA-mPEG *hyaluronic acid-methoxy Poly(ethylene-glycol) amine* (Di)methyl maleate bond: PDLLA-PEG *Poly(D,L-lactide)-Poly(ethylene-glycol)*	[20,21]
Redox potential	Disulfide bond	MPEG-P(BHD-SS)-MPEG *Poly(ethylene-glycol)-b-polycarbonate-Poly(ethylene-glycol)*	[22]
Temperature	Lower critical solution (LCST) Upper critical solution (UCST)	LCST: PNIPAM *Poly-N-isopropylacrylamide* UCST: iMAPA *Insoluble multi-L-arginyl-poly-L-aspartic*	[23,24]
Light	Photo-triggered groups	Polydopamine	[25,26]
Ultrasound	Disulfide bond Particles aggregates	PLA-S-S-PEG *Poly(L-lactide)-S-S-Poly(ethylene-glycol)* PLGA aggregates *Poly(lactic-co-glycolic acid)*	[27,28]
Magnetism	Incorporation of magnetic particles	Iron nanoparticles	[29,30]
Shear stress	Flexible particles, generally hydrogels	ADEN/THYM polymersomes *Adenine/thymine functionalized block co polymers*	[31]

**Table 2:** Cleavable pH-responsive bonds.

Cleavable Bond	pH
Imine	<5–7
Hydrazone	<5
Hydrazide	<5
Oxime	<5
(di)Methyl maleate	<6.8

**Table 3:** GSH level for different cellular environments.

Environment	GSH Level
Intracellular	1–10 mM
Extracellular	1–10 μM
Brain Cancer	0.5–3 mM

**Table 4:** A summary of recent multiple responsive polymers used for particle drug delivery systems (PDDS).

Polymer	Stimuli	Description	Ref.
PMAEFc-ONB-PDMAEMA *poly(2-methacryloyloxyethyl ferrocenecarboxylate)-(5-propargylether-2-nitrobenzyl bromoisobutyrate)-poly(di-methylaminoethyl methacrylate)*	Light pH Temperature Redox: -oxidative -reduction	pH-responsive and LCST: PDMAEMA. Oxidation/reduction: ferrocenyl groups. Light responsive: o-nitrobenzyl methyl esters.	[104]
Poly[HBCEEM-b-(NIPA-r-PEGMA)] (PHNP) *2-(2-((4-(hexyloxy)benzyloxy)carbonyl)ethylthio)ethyl acrylate, N-isopropyl acrylamide, poly(ethylene glycol methyl ether acrylate)*	pH Temperature Redox	pH-responsive: HBCEEA. Disulfide bond (S-S): redox responsive. Temperature-responsive: NIPA and PEGMA.	[105]
Fc-DEAE-AM *poly(2-(3-(N-(2-(diethylamino)ethyl)acrylamido)-propanoyloxy)ethyl ferrocenecarboxylate)*	Redox pH CO_2_	Redox-responsive: Fc. pH-responsive and CO_2_: DEAE.	[106]
PDA *Polydopamine*	Light pH Redox (if S-S)	NIR-responsive and pH: dopamine. Redo-responsive: incorporation of disulfide bond (S-S).	[25,88,89,92,107,108]
P(MEO_2_MA-co-OEGMA)-b-P(MAA-co-SPMA) *Poly(2-(2-methoxyethoxy)ethylmethacrylate-co-oligo(ethylene glycol) methacrylate)-block-poly(methac*r*cid-co-spiropyran methacrylate)*	pH Light Temperature	UV light-responsive: SP-MC. pH-responsive: P(MAA-co-SPMA). LCST: change based on monomer ratio.	[109]
PSB-block-P(NIPAM-A)) *poly(sulfobetaine)-b-poly(N-isopropylacrylamide-co-dopamine methacrylamide)* iMAPA-HA *insoluble Multi-L-arginyl-poly-L-aspartate- hyaluronic acid* 700DX-P(NIPAAm/AIPAAm-PMM) *poly(N-isopropylacrylamide)* *-2-aminoisoprpylacrylamide-2-propionic-3-methyl-maleic* PAA@PHEMA *poly(acrylic acid)-poly(2-hydroxyethylmethacrylate)* PBM-b-ND *poly(butyl methacrylate)-b-poly(N-isopropylacrylamide-co-N,N-dimethylacrylamide)* PMAA-b-PNIPAM *poly(methacrylic acid)-b-poly(N-isopropylacrylamid* poly(NIPAM-co-GMA) *poly(N-isopropylacrylamid)-co-glycidyl methacrylate*	Temperature pH	Thermo-responsive (LCST): NIPAM. Thermo-responsive (UCST): iMAPA, combined with pH-responsive block: -poly(acrylic acid) PAA, -metal–catecholate, -iMAPA, -N-alkyl groups, -PDPA, -hydrazine units.	[78,110,111,112,113,114,115]
HA-VE and PBAEss *hyaluronic acid-vitamine E* *poly(β-amino ester)* mPEG-P(TPE-co-AEMA) *poly ethylene glycol-poly(tetrapheny-lethene-co-2-azepane ethyl methacrylate)* HA-SH-CS *thiol-hyaluronic acid-chitosan* PPZ *Polyphosphazene* PEG modified trimethyl chitosan *Polyethylene glycol-trimethyl chitosan* PAE(-ss-mPEG)-g-Chol *poly(-amino ester)-g-poly(ethylene glycol) methyl ether-cholesterol* PEG-SS-CPT *Polyethylene glycol- disulfide bond- camptothecin*	pH Redox	Redox-responsive: disulfide bond (S-S). pH-responsive segments: -poly(β-amino ester), -(PAEMA): pH > 6.8 hydrophobic, pH < 6.8 hydrophilic, -polyelectrolyte complexes, -cross-linked polyphosphazene, -trimethyl chitosan, -copolymer poly(-amino ester)-g-poly(ethylene glycol) methyl ether-cholesterol.	[116,117,118,119,120,121,122]
PEG-PEI-GEM *polyethylenimine-graft-poly(ethylene glycol)- gemcitabine*	pH Light	Light-responsive: photo-cleavable-o-nitrobenzyl, with a linker of thermosensitive: PEG–PEI.	[123]
PEO-PEtG-PEO *Poly(ethyl glyoxylate)-Poly(ethylene oxide)*	Light (UV) Redox	Redox-responsive: disulfide bond (S-S). Light-responsive: o-nitrobenzyl moiety.	[124]
BU-PPG *Uraciland-oligomeric polypropylene glycol*	Temperature Light	Light-responsive: uracil. Thermoresponsive: oligomeric PPG.	[103]
pDHPMA-DOX *poly[N-(1, 3-dihydroxypropyl) methacrylamide]-doxorubicin*	pH Enzyme	Enzyme-responsive: Gly–Phe–Leu–Gly (GFLG), with a linker of pH-responsive: hydrazone bond.	[125]
